# Investigating the Mediating Role of Distress Between Nomophobia and Student Mindfulness: A Cross-Sectional Study

**DOI:** 10.3390/healthcare13192512

**Published:** 2025-10-03

**Authors:** Badr Alnasser, Rakesh Kumar

**Affiliations:** Department of Health Management, College of Public Health and Health Informatics, University of Ha’il, Ha’il P.O. Box 2440, Saudi Arabia

**Keywords:** nomophobia, student’s mindfulness, distress, mediation analysis, Saudi Arabia

## Abstract

Background/Objectives: In the age of digitalization, nomophobia has emerged as a relevant issue, especially among university students who utilize smartphones heavily for academic and social purposes. The Stressor–Strain–Outcome (SSO) framework explains the relationship between stressors, strain, and outcomes. Stressors such as nomophobia induce psychological strain. This strain subsequently influences outcomes like mindfulness. Nomophobia has been linked to higher distress, including depression, anxiety, and stress, that can negatively impact students’ focus. However, the mechanisms by which nomophobia impacts mindfulness remain less explored. Hence, this study aims to analyze the mediating effect of distress on the relation between student’s nomophobia and mindfulness. Methods: In this quantitative study, the researcher employed a structured close-ended survey to collect data from 723 students at the University of Ha’il in Saudi Arabia. Nomophobia was measured using the Nomophobia Questionnaire (NMP-Q). The level of distress was measured using the Depression, Anxiety, and Stress scale (DASS-21) Furthermore, the assessment of mindfulness was conducted using the Mindful Attention Awareness Scale (MAAS). Structural equation modeling was utilized to test the hypotheses of this study. Results: The results from PLS-SEM indicate that nomophobia did not directly reduce mindfulness, as its effect was statistically non-significant (β_1 = −0.052, *p*-value = 0.168). This suggests that nomophobia alone may not weaken focus. However, it significantly increased distress, particularly depression (β_2a = 0.327, *p*-value < 0.001), anxiety (β_2b = 0.294, *p*-value < 0.001) and stress (β_2c = 0.259, *p*-value < 0.001). In plain terms, students with higher nomophobia reported more depression and stress, which in turn reduced mindfulness. Anxiety, however, did not significantly affect mindfulness (β_3b = 0.006, *p*-value < 0.933), indicating its influence may be negligible or context-specific. Mediation analysis confirmed indirect effects of nomophobia on mindfulness through depression (β_4a = −0.096, *p*-value < 0.001) and stress (β_4c = −0.045, *p*-value < 0.020). Together, these mediators explained a substantial portion of the variance in mindfulness. Conclusions: The findings align with the SSO model, indicating that nomophobia acts as a stressor, exacerbating distress, which in turn reduces mindfulness. From a practical perspective, the results highlight the need for comprehensive student support. Universities should integrate digital wellness programs, stress-management resources, and mindfulness training into their services. Limitations and Future Research: The cross-sectional design and convenience sampling restrict causal inference and generalizability. Future studies should employ longitudinal research designs. They should also examine diverse cultural contexts. In addition, researchers should investigate potential mediators such as social support and sleep quality.

## 1. Introduction

The student mindfulness is the ability to remain present and aware of the academic task and to effectively regulate distractions. Mindfulness is defined as a non-judgmental awareness of the present moment. It is widely recognized as a protective factor for student well-being and academic performance [1,2,3]. Elevated levels of psychological distress, including depression, anxiety, and stress, are common in higher-education contexts. These conditions may explain how digital stressors affect students’ ability to sustain attention. Student mindfulness is being present in the moment, regulating emotions, and being capable of sustaining concentration, which is critical to learning and academic achievement [1]. Mindfulness is important in educational setting as it improves cognitive functioning, enhances information retention, and creates a positive learning atmosphere [2]. Mindful students have better academic abilities to deal with academic problems, manage anxiety, and attain educational objectives [3,4]. Despite this, it is becoming increasingly hard to maintain concentration with the prevalence of smartphones and the problem of nomophobia.

Nomophobia is referred to as the phobia of being without a mobile phone [5]. Nomophobia is becoming increasingly common among students. Nomophobia is characterized by anxiety, discomfort, or distress away from one’s smartphone [6]. Longitudinal research indicates that mindfulness predicts both GPA (Grade Point Average) and student retention. Cross-cultural studies further demonstrate its buffering role against stress in diverse populations [7,8]. Understanding how nomophobia influences mindfulness, particularly in student populations, is therefore essential.

We adopt the SSO framework in this study. The framework suggests that external stressors, such as nomophobia, intensify internal strain including depression, anxiety, and stress. This strain subsequently results in negative outcomes, such as reduced mindfulness [9]. Prior research has validated this model in the context of workplace stress [10]. However, its application to nomophobia and mindfulness in student populations remains underexplored. Stress, on the other hand, is a physical and emotional response to a perceived difficulty, which negatively affects memory, focus, and decision-making [11]. While these issues vary, they have a tendency to co-occur and support one another, sustaining a cycle that also erodes student focus.

This study was conducted among students at the University of Ha’il in Saudi Arabia. The university context is characterized by high smartphone penetration and rapidly expanding higher-education enrollment. Cultural norms related to family expectations, gender roles, and academic competition may uniquely shape the manifestation of distress. These norms can also influence how distress affects attentional outcomes. By situating our research in this context, we contribute to the growing body of cross-cultural literature. We also extend the application of the SSO model beyond Western samples.

Existing studies often examine the outcomes of nomophobia in relation to performance or social well-being. However, they rarely integrate mindfulness as an academic resource. In addition, few studies distinguish the specific roles of depression, anxiety, and stress as mediators. By applying the SSO model, this study addresses these gaps. It further clarifies the psychological mechanisms through which nomophobia reduces mindfulness in students.

### 1.1. Conceptual Framework and Hypothesis

**Figure 1:**
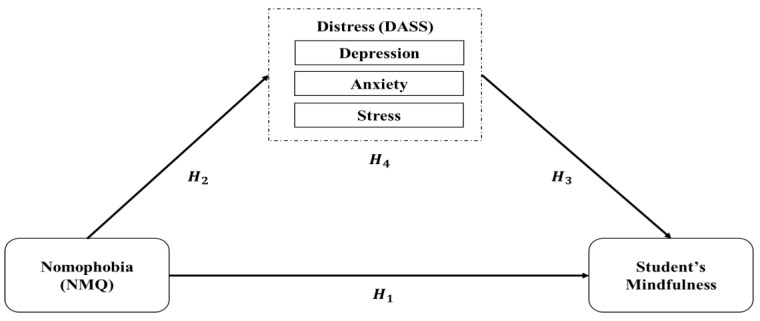
Research Model.

Guided by the SSO model, we conceptualize nomophobia as the stressor. Psychological distress, including depression, anxiety, and stress, represents the strain. Student mindfulness serves as the outcome. Within this framework, external pressures heighten internal distress, which in turn reduces mindfulness in academic contexts as proposed by Koeske and Koeske [12]. Figure 1 depicts this logic and the hypotheses we test.

#### 1.1.1. Nomophobia and Student Focus

Nomophobia has also been connected with poor cognitive functioning and concentration among students. Overuse of smartphones has been found to lead to distractions, shorter attention spans, and failure to maintain focus on study material [1]. For instance, a study by Aldhahir, M. [13] indicated that students who had greater levels of nomophobia were more susceptible to study disruption brought about by the overuse of smartphones. Similarly, a study by Al Ali and Matarneh [14] indicated that smartphone addiction was found to be negatively related to academic performance. Therefore, it is likely that nomophobia interferes with the concentration of the students. These findings stipulates that the greater the extent of nomophobia, the more it undermines the concentration of the students. Some studies, however, also noted that the relationship between nomophobia and concentration is not necessarily direct, and mediating variables such as distress may have a role to play [15,16]. Therefore, we hypothesized that nomophobia has a negative direct effect on student mindfulness (H1).

#### 1.1.2. Nomophobia and Distress

The relationship between nomophobia and psychological distress is highly documented in the literature. Nomophobia is also associated with elevated anxiety, depression, and levels of stress, particularly among students and youth [17]. For example, Alwafi, Naser [18] revealed that those with higher levels of nomophobia had significantly higher levels of depression and anxiety symptoms. Another research study by Arpaci, Baloğlu [1] revealed that nomophobia was a significant predictor of university students’ stress. This finding suggests that elevated levels of nomophobia lead to more distress among students. Some of the mechanisms behind the relationship could include the ever-present need to remain connected and the need to remain socially engaged online [19,20,21]. Therefore, we hypothesized that higher nomophobia is associated with greater distress (H2). The distress includes depression, anxiety, and stress.

#### 1.1.3. Distress and Student Focus

Research has shown that depression, anxiety, and stress adversely affect cognitive abilities and hinder academic achievement. Depression, for instance, correlates with poor concentration, memory, and decision-making, which are all vital to academic performance [22]. Anxiety also leads to shorter attention spans and poor cognitive function, particularly in stressful academic environments [10]. Stress, another key element of distress, also negatively influences students’ ability to concentrate and retain information [11]. These findings assert that a rise in distress leads to a harmful impact on the concentration of the students. Literature also identifies the fact that distress could end up being a barrier to effective learning and academic performance [23,24]. These findings highlight the need to address these psychological issues in learning environments. Therefore, we hypothesized that higher distress is associated with lower mindfulness (H3). Again, the distress means depression, anxiety, and stress here.

#### 1.1.4. Mediating Role of Distress

The mediating effect of distress has also been explored in another research. Zhou, Chen [25] for example, found that anxiety and depression mediated the effect of poor cognitive performance caused by problematic smartphone use. Another study by Wang, Li [26] found that the effect of poor academic performance caused by smartphone dependency was partly mediated by stress. These findings show that the effect of nomophobia on the concentration of students is significantly mediated by distress. This research found that it is likely that nomophobia lacks a direct effect on concentration but worsens psychological distress, which impairs cognitive functioning and academic performance. The requirement to deal with distress as a primary issue to avoid the effects of nomophobia among students is therefore of great significance. Therefore, we hypothesized distress mediates the effect of nomophobia on mindfulness (H4).

Past research has indicated that nomophobia is connected to decreased concentration among students, increased psychological distress, and impaired academic performance [13,22,27]. The literature also identifies the role of distress as a mediator in the nomophobia and concentration. For example, the elimination of distress could potentially negate the negative impacts of smartphone addiction [28]. Expanding upon previous research, this study aims to better understand the mechanisms by which nomophobia is connected to concentration among students. The research informs the development of intervention strategies to increase mindfulness and reduce distress among students. Figure 1 presents the conceptual framework of this research which is based on SSO model and the mechanism drawn from prior empirical works. As shown in Figure 1, we estimate the direct path from nomophobia to mindfulness (H1). We also specify three indirect paths (H4a–H4c), following the SSO sequence from stressor to strain to outcome. This approach clarifies the role of each mediator. It also ensures that the conceptual model aligns with the subsequent analysis and reporting.

Nomophobia (stressor) is hypothesized to increase depression, anxiety, and stress (strain; H2a–H2c). These distress factors are expected to reduce mindfulness (outcome; H3a–H3c). A dashed path represents the hypothesized direct effect of nomophobia on mindfulness (H1). H4a–H4c indicate the indirect, mediated effects of nomophobia on mindfulness through each distress component. Measurement details are provided in Section 2.3.

### 1.2. Goals of This Study

This research aims to investigate the effect of nomophobia on student mindfulness. The study particularly focusses on how distress like depression, anxiety, and stress—mediate this relationship. Using the quantitative method, the study tries to measure and compare the strength and significance of the relationships through the application of statistical modeling. Empirical evidence provides insights into the psychological mechanisms that exist between mobile device dependency and the mental state and academic performance of students. Ultimately, this research intends to aid in formulating evidence-based approaches aimed at fostering mindfulness and mitigating distress among students.

## 2. Materials and Methods

### 2.1. Research Design

This study utilizes a quantitative, cross-sectional survey design to examine the relationship between nomophobia, distress (anxiety, depression, and stress), and student mindfulness. The study is informed by a positivist philosophy. It focuses on the use of objective measurement and statistical inference to test a hypothesis and make findings that are generalizable [29]. A deductive reasoning is followed, with a known theory as the framework (e.g., the SSO model), to formulate hypotheses, which are later tested with empirical evidence [30]. Data is collected through structured close-ended questionnaires. Advanced statistical methods, including structural equation modeling (SEM), are also employed to investigate relationships between variables. The quantitative method is suitable as it allows for precise measurement, hypothesis testing, as well as the ability to generalize findings to a population level [31]. The design is rigorous, reliable, and valid in the accomplishment of the research objectives.

### 2.2. Sample Size Determination and Power Analysis

Beyond the 10-times heuristic [32], we estimated the minimum sample size required for the most demanding regression. This regression included mindfulness as the dependent variable, with nomophobia, three mediators, and demographic controls as predictors. Using G*Power 3.1 [33] α = 0.05, power = 0.80, and small effect sizes (f^2^ = 0.02), the required sample was approximately 400. Our achieved sample of 723 therefore provides ample statistical power, even under conservative assumptions [34].

### 2.3. Setting and Participants

This cross-sectional study was conducted at the University of Ha’il (UoH) in Saudi Arabia, a large public university with multiple colleges (e.g., Health, Sciences, Arts, Business). The sampling frame included currently enrolled undergraduate and graduate students across faculties. Using convenience sampling, we obtained 723 responses. Convenience sampling may over- or under-represent certain groups, such as digitally engaged students. This limitation restricts the generalizability of findings beyond similar public university contexts in Saudi Arabia and the region. We acknowledge this constraint and discuss its implications accordingly [15]. Demographic summaries appear in Table 1.

#### 2.3.1. Eligibility Criteria

Eligibility criteria included: age ≥18 years, current enrollment at UoH (undergraduate or graduate), regular smartphone use, and provision of informed consent. No exclusions were applied for exchange student status or self-reported mental health history. The study focused on non-clinical, self-report measures and did not involve diagnostic screening. Incomplete responses and cases that failed attention checks were excluded.

#### 2.3.2. Recruitment and Data Collection Procedure

Data were collected via a self-administered survey during February–April 2025. The survey was distributed online through institutional email and LMS links, and offline via QR codes on campus posters and paper forms in large lecture classes. Participation was voluntary, anonymous, and uncompensated. To mitigate response bias and common method variance, we assured participants of confidentiality and emphasized the absence of right or wrong answers. We also separated predictors and outcomes into distinct sections and randomized item order within each scale [32]. Attention check items were embedded, and submissions failing these checks or completed unrealistically quickly were removed.

### 2.4. Ethical Consideration

This study follows rigorous ethical protocols to uphold integrity, confidentiality, and participant well-being. Informed consent is secured from all participants, providing a clear explanation of the study’s objectives, procedures, and their unrestricted right to withdraw at any stage without repercussions. To preserve confidentiality and anonymity, no personally identifiable data is captured, and all data is kept securely in password-protected systems. Respondents are assured that the answers they give will solely be used for research and will not be revealed to external groups. The study also avoids causing harm or discomfort by using non-intrusive approaches and providing mental health resources to those who feel uncomfortable. The Research Ethics Committee (REC) at the University of Ha’il granted ethical approval for this study dated 20 January 2025, No. H-2025-573, to ensure compliance with ethical standards.

### 2.5. Measures

The study employs validated scales to measure the key variables: nomophobia as independent variable, distress as mediator, and student mindfulness as the dependent variable. Nomophobia is evaluated using the 20-item Nomophobia Questionnaire (NMP-Q) Yildirim and Correia [35]. It assesses the fear of being without a mobile phone across four dimensions: communication difficulties, loss of connectedness, restricted access to information, and reduced convenience. Items are rated on a 7-point Likert scale (1 = strongly disagree to 7 = strongly agree), with higher scores indicating greater levels of nomophobia.

Distress, encompassing depression, anxiety, and stress, is measured using the DASS-21 scale as developed by Lovibond and Lovibond [36]. It is a 21-item instrument divided into three subscales, each containing seven items. Responses are rated on a 4-point Likert scale (0 = did not apply to me at all to 3 = applied to me very much or most of the time). Higher scores indicate greater severity of symptoms.

Student mindfulness is assessed using the 15-item Mindful Attention Awareness Scale (MAAS), developed by Carlson and Brown [37]. It was designed to measure present-moment awareness and attention. Items are rated on a 6-point Likert scale (1 = almost always to 6 = almost never), with higher scores reflecting greater dispositional mindfulness. All the measures utilize Likert-type responses for greater consistency and easy data analysis. The measures have been chosen on the grounds of being reliable, valid, and most commonly used in available research to ensure strong and accurate measurement of the constructs.

All constructs were measured with well-validated instruments: NMP-Q for nomophobia, DASS-21 for depression, anxiety, and stress, and MAAS for mindfulness. Reliability and validity results are reported in the results section. These results provide evidence of cultural adaptation and psychometric soundness [32].

### 2.6. Analysis

Analyses were conducted in Smart-PLS 4 using PLS-SEM. The procedure involved two stages: measurement model evaluation and structural model assessment. Bootstrapping was applied to generate statistical inference [32].

#### 2.6.1. Rationale for PLS-SEM vs. CB-SEM

We selected PLS-SEM to predict mindfulness and to estimate mediated effects within a model containing multiple latent variables. The model also included a second-order construct structure for nomophobia (NMP-Q dimensions). PLS-SEM is robust to non-normal data, manages model complexity efficiently, and emphasizes maximizing explained variance in endogenous constructs [32]. We report global fit indices (e.g., SRMR, NFI) for completeness and transparency. However, we emphasize prediction-oriented criteria and effect sizes.

#### 2.6.2. Measurement Model Evaluation Criteria

We assessed indicator reliability using loadings ≥ 0.70. Internal consistency was evaluated through Cronbach’s α and CR, both expected to exceed 0.70. Convergent validity was established with AVE ≥ 0.50. Discriminant validity was examined using HTMT (≤0.90) and the Fornell–Larcker criterion, which requires the square root of AVE to exceed inter-construct correlations [32].

#### 2.6.3. Structural Model Evaluation and Inference

We examined collinearity using inner VIF values, with thresholds set below 3.3. Path coefficients were assessed with bootstrapped 95% confidence intervals (resamples = 5000). We also reported effect sizes (f^2^), explained variance (R^2^), and predictive relevance (Q^2^). Model-level fit was evaluated using SRMR (<0.08) and NFI (approximately ≥0.90). Indirect effects were tested with bootstrapped confidence intervals. In cases where direct effects were non-significant but indirect effects were significant; we explicitly described the pattern as “indirect-only” (complete) mediation [32].

#### 2.6.4. Common Method Bias Checks

To address common method bias, we applied procedural remedies such as confidentiality assurances, scale separation, and randomized item order. We also conducted statistical checks. Harman’s single-factor test showed that no single factor accounted for the majority of variance. This result suggests that common method bias was not a severe threat [32].

#### 2.6.5. Control Variables

To reduce omitted variable bias, we included demographic controls as covariates predicting mindfulness. These controls comprised gender, age group, program level, daily smartphone use hours, and living situation. We also tested a robustness model in which the controls predicted the three mediators. The conclusions remained unchanged.

#### 2.6.6. Correlations Among Mediators

Depression, anxiety, and stress were modeled as parallel mediators. Their latent correlations naturally arise in PLS-SEM via shared predictors (nomophobia). Inter-construct correlations are reported in results section while avoiding reciprocal paths between mediators to preserve interpretability of mediation effects [32].

## 3. Results

### 3.1. Demographic Analysis

In total, response was collected from 723 students. Table 1 depicts the demographic details of the participants. From these 723 participants, 452 were male (representing 62.5%), and 271 were female (representing 37.5%). Additionally, 625 (86.44%), and 98 (13.6%) of participants are studying in bachelor’s and master’s programs. Furthermore, 473 (65.4%), 116 (16%), 62 (8.6%), and 72 (10%) of the participants represents the age category of 18–21, 22–25, 26–30, and 31 or above, respectively. Moreover, 19 (2.6%), 171 (23.7%), 271 (37.5%), and 262 (36.2%) participants uses mobile phone 1–2, 3–4, 4–6, and 7–8 h, respectively. Finally, 10 (1.4%), 586 (81.1%), 52 (7.2%), and 75 (10.4%) participants lives on campus hostel, off-campus with family, off-campus with roommates, and alone, respectively.

### 3.2. Descrptive Statistics

Table 2 presents descriptive statistics for the study variables. Students reported elevated levels of nomophobia (M = 96.4, SD = 22.7), with 35% scoring in the severe range. Depression levels were also notable (M = 13.8, SD = 8.2), with 18% classified as severe. Anxiety scores averaged 12.9 (SD = 7.6), with 22% in the severe range. Stress scores averaged 15.5 (SD = 8.9), with 14% in the severe range. These results indicate substantial psychological distress in the sample. Mindfulness scores were moderate (M = 3.58, SD = 0.74), reflecting variability in students’ attentional awareness.

### 3.3. Convergent Validity and Reliability

To ensure the adequacy of the measurement model, convergent validity, reliability, and discriminant validity were evaluated in an integrated manner. Table 3 demonstrates the convergent validity and reliability of the measured constructs of COMM, CONN, INFO, CONV, depression, anxiety, stress, and mindfulness. Convergent validity was estimated with the use of factor loading (FL), Cronbach alpha (CA), composite reliability (CR), and average variance extracted (AVE). The constructs all demonstrated high reliability with FL exceeded 0.70, CA > 0.70 and with CR values exceeding 0.70, as suggested by Hair and Alamer [32]. AVE meeting the 0.50 cut-off value as suggested by Fornell and Larcker [38]. Therefore, establishing convergent validity. The measurement model’s validity and reliability confirm it for subsequent analysis.

### 3.4. Discriminant Validity and Reliability

Table 4 presents HTMT ratios to test for discriminant validity between constructs: Anxiety (ANXI), Communication (COMM), Connectedness (CONN), Convenience (CONV), Depression (DEPR), Information (INFO), Mindfulness (MIND), and Stress (STRS). All HTMT values are less than the recommended cut-off value of 0.90 [39]. Therefore, it confirms the presence of discriminant validity. These results confirm that the constructs are distinct and reliable for further analysis.

According to the Fornell and Larcker [38] criterion, the square root of AVE (diagonal values) of each construct must be higher than the correlations of a construct with the other constructs (Table 5). As the diagonal values of all the constructs are higher than the off-diagonal correlations. Therefore, it confirms the constructs’ reliability and distinctiveness, reinforcing the measurement model’s validity.

HTMT ratios were below 0.90, confirming discriminant validity among constructs. The Fornell–Larcker results (Table 4) further supported this conclusion. For every construct, the square root of AVE (diagonal) exceeded its correlations with other constructs. These findings indicate that each construct is empirically distinct.

### 3.5. Model Fit

Once reliability and validity were confirmed, the overall model fit was evaluated. Table 6 provides the model fit indices for both the saturated and estimated models. The SRMR value of 0.052 is lower than the suggested 0.08 threshold, signifying a well-fitting model [40]. The chi-square value is 6084.242, which is typically sensitive to sample size but is reported for completeness. With a value of 0.890, the NFI is near the acceptable 0.90 threshold, reflecting an adequate model fit [41]. The discrepancy measures (d_ULS = 4.272 and d_G = 1.586) are also provided, though they lack specific thresholds. Overall, the SRMR and NFI values confirm that the model fits the data well, strengthening its validity for subsequent analysis. These indices confirm that the model demonstrated a satisfactory overall fit, supporting its use for hypothesis testing.

**Figure 2:**
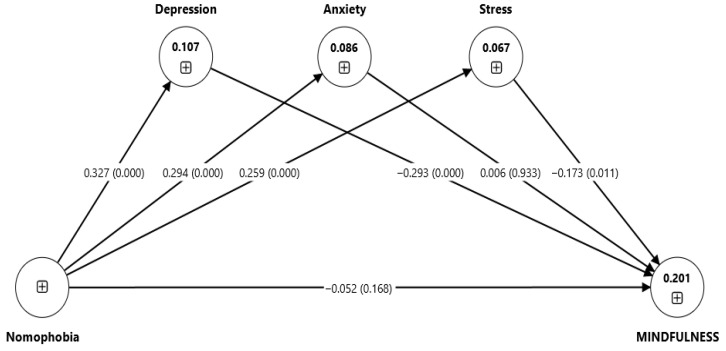
SEM Model.

Figure 2 presents the structural model with standardized path coefficients. Values in parentheses represent t-values derived from bootstrapping. The figure has been redrawn at higher resolution to enhance clarity.

### 3.6. Hypothesis Testing

Following confirmation of model fit, hypothesis testing was conducted using bootstrapping with 5000 resamples to estimate path coefficients, *p*-values, and confidence intervals. Table 7 reveals hypothesis testing for the effects of nomophobia on student mindfulness, mediated by distress (depression, anxiety, and stress). The results from PLS-SEM indicates that nomophobia showed no significant effect on mindfulness (β_1 = −0.052, *p*-value = 0.168, 95% CI [−0.12, 0.03]) rejecting H1. Additionally, nomophobia significantly increased distress, particularly depression (β_2a = 0.327, *p*-value < 0.001, 95% CI [0.25, 0.41]), anxiety (β_2b = 0.294, *p*-value < 0.001, 95% CI [0.20, 0.38]) and stress (β_2c = 0.259, *p*-value < 0.001, 95% CI [0.15, 0.35]), supporting the study’s hypotheses H2a, H2b, and H2c. It confirms that higher levels of nomophobia increase distress among students. Moreover, while examining the direct effects of distress factors on mindfulness, depression (β_3a = −0.293, *p*-value < 0.001, 95% CI [−0.37, −0.21]) and stress (β_3c = −0.173, *p*-value < 0.011, 95% CI [−0.30, −0.04]) were found to have significant negative impacts, supporting H3a and H3c. In contrast, anxiety did not significantly affect mindfulness (β_3b = 0.006, *p*-value < 0.933, 95% CI [−0.09, 0.10]), resulting in the non-acceptance of H3b.

The mediation analysis further revealed notable indirect effects. Nomophobia negatively affected mindfulness through depression (β_4a = −0.096, *p*-value < 0.001, 95% CI [−0.14, −0.06]) and stress (β_4c = −0.045, *p*-value < 0.020, 95% CI [−0.08, −0.01]), supporting H4a and H4c. These results indicate that depression and stress completely mediate the connection between nomophobia and mindfulness, since the direct influence of nomophobia on mindfulness was insignificant. This suggests that the negative impact of nomophobia on mindfulness operates entirely through the mechanisms of increased depression and stress. However, the indirect effect through anxiety was not significant (β_4b = 0.002, *p*-value < 0.934, 95% CI [−0.04, 0.05]) rejecting H4b. Taken together, these findings indicate that depression and stress completely mediated the relationship between nomophobia and mindfulness, while anxiety did not function as a mediator. The direct path from nomophobia to mindfulness was not significant. However, the indirect paths through depression and stress were significant, indicating indirect-only (complete) mediation. Anxiety did not significantly mediate the nomophobia–mindfulness relationship. This may reflect contextual factors. In our sample, anxiety levels were high but not strongly linked to attentional regulation. Cultural norms around academic stress or adaptive coping strategies may buffer its effect.

Overall, Depression and stress explained 20.1% of the variance in mindfulness (R^2^ = 0.201). Effect sizes were moderate for depression (f^2^ = 0.12) and stress (f^2^ = 0.08). In contrast, effect sizes for nomophobia (<0.01) and anxiety (<0.01) were negligible.

## 4. Discussion

### 4.1. Summary of Findings

The findings of this study offer significant insights into the relationships between nomophobia, distress, and the mindfulness of students. The findings support the hypothesis that the increase in nomophobia elevates the level of distress among students, primarily depression, anxiety, and stress (H2a, H2b, H2c). The direct impact of nomophobia on the mindfulness of students (H1), however, was not significant, i.e., that nomophobia in isolation does not interfere with concentration. Instead, the study found that factors of distress, i.e., depression and stress, fully mediate the negative effect of nomophobia on the mindfulness of students (H4a, H4c). Anxiety did not have an effect as a mediating variable, however (H4b). This suggests that anxiety may operate differently than depression and stress. Its influence appears more context-dependent or normalized in academic settings. In Saudi Arabia, competitive pressures and high levels of academic anxiety are particularly common. These results support the SSO model, i.e., that nomophobia is a stressor that intensifies distress, which in turn decreases the level of mindfulness. The results also highlight the necessity to target the process of distress as the primary way through which nomophobia impacts the psychological and academic performance of students.

### 4.2. Comparison with Prior Research

The findings of the present study corroborate and extend existing research on nomophobia and its psychological impact. For instance, Alwafi, Naser [18] concluded that nomophobia was a significant predictor of anxiety and stress, a finding that the present study corroborated. Kumar, Yousif [42] also found that nomophobia significantly affects distress (stress, anxiety, and depression). Similarly, Karila, Scher [6] indicated that compulsive smartphone use was associated with more depression and anxiety, a finding that also supports the mediating effect of the distress factors. Still, unlike current research that generally explored direct effects, the current study confirms the full mediation of the nomophobia–mindfulness relation by distress factors, providing a better understanding of the mechanisms involved.

The non-significant direct correlation between nomophobia and mindfulness contradicts another research. For example, Paterna, Alcaraz-Ibáñez [43] found a direct inverse correlation between smartphone use and academic performance with a direct impact on concentration. Our findings show that the direct effects disappear once distress is included as a mediator. This suggests that depression and stress fully absorb the explanatory power of nomophobia. The inconsistency may have arisen due to the use of varying measurement tools or the sample characteristics. The finding that anxiety did not mediate the correlation between nomophobia and mindfulness also contrasts with research like that of Zhou, Chen [25], which established anxiety to mediate significantly. This inconsistency may be explained by differences in measurement instruments, such as generalized anxiety scales versus academic-context scales. Cultural norms and gender ratios within samples may also moderate the anxiety–mindfulness relationship.

The use of the SSO framework adds explanatory depth. Alternative models, such as the Technology Acceptance Model or Cognitive–Behavioral frameworks, emphasize motivations or usage patterns. In contrast, the SSO framework clarifies a processual mechanism: nomophobia → distress → reduced mindfulness. By highlighting this mediation pathway, the current study provides theoretical precision. It explains why interventions that target distress, rather than technology use itself, may be more effective.

### 4.3. Theoretical Implications

The study also moves the field forward by validating the use of the SSO model in nomophobia. The model was used in prior research to study technology use and job stress, but its use with students and with the domain of mindfulness is new. It expands the theoretical foundation of how digital stresses like nomophobia impact psychological and academic functioning. Specifically, the finding that depression and stress—but not anxiety—mediate the pathway underscores the need to distinguish forms of emotional strain. Somatic-cognitive states such as depression and stress directly drain attentional resources. In contrast, arousal states like anxiety may not consistently reduce mindfulness, depending on context. This boundary condition suggests the need to refine the SSO model for digital stressors.

### 4.4. Practical Implications

The findings carry practical relevance for multiple stakeholders. For universities, digital wellness programs, mindful smartphone-use workshops, and stress-management curricula should be integrated into student support. For educators, mindfulness practices such as brief attention or breathing exercises can be embedded into classroom routines to buffer against distress. For policymakers, priorities include expanding campus mental health services, supporting awareness campaigns on technology overuse, and fostering balanced digital environments. For students, recommended strategies include self-monitoring (e.g., screen-time tracking, scheduled breaks), adaptive coping (e.g., exercise, peer support), and maintaining sleep hygiene. By targeting distress—particularly depression and stress—these measures may help mitigate the academic harms of nomophobia.

### 4.5. Limitations and Future Directions

Despite its contributions, the study also has a number of shortcomings. First, the use of self-report measures could lead to response bias, as the participants could over- or underestimate the experience of nomophobia, distress, and mindfulness. Second, the study’s cross-sectional design means that causal links between the variables cannot be determined. Longitudinal studies are needed to see how the relationships change over the long term. Third, the sample, whilst large, was limited to university students, potentially reducing the generalizability of the findings to other groups, such as working professionals or younger adolescents. Fourth, the study controlled for the individual distress variables (depression, anxiety, and stress) but not for other potential mediators such as social support or sleep quality. Finally, the data were collected in a single geographic location, which reduces the cultural generalizability of the findings. Future studies should address these shortcomings by employing heterogeneous samples and longitudinal research designs. They should also incorporate additional mediating variables. Expanding comparative analyses with non-regional studies, such as European and North American research on smartphone addiction, nomophobia, and mindfulness, will further strengthen the external validity of these findings.

## 5. Conclusions

This study demonstrates that nomophobia undermines student mindfulness indirectly by elevating psychological distress. Depression and stress emerged as complete mediators in this process. In contrast, anxiety did not significantly predict mindfulness, suggesting boundary conditions within the stressor–strain–outcome framework. These findings extend the SSO model to student mindfulness and clarify the emotional mechanisms through which digital dependence impairs attentional resources.

The findings highlight actionable opportunities for higher education institutions and policymakers. Universities should invest in digital wellness programs, mindfulness training, and targeted mental-health services to reduce student distress. Faculty can also embed short mindfulness exercises into teaching to help mitigate stress and depression. The findings carry theoretical implications as well. They underscore the need to refine the SSO framework by accounting for differential mediator effects. Depression and stress appear consistently impactful, whereas anxiety may exert context-dependent influence.

Future research should extend beyond a single cultural and institutional setting, test additional mediators (e.g., social support, sleep quality), and adopt longitudinal or experimental designs to establish causal mechanisms. Comparative cross-cultural research will strengthen the generalizability of this line of work. Integration with non-regional smartphone addiction literature will also enhance its international relevance.

## Figures and Tables

**Table 1:** Demographic Characteristics of the Participants.

Characteristics	Categories	N	%
Gender	Male	452	62.5%
	Female	271	37.5%
Education	Bachelor’s Program	625	86.44%
	Master’s Program	98	13.6%
Age (years)	18–21	473	65.4%
	22–25	116	16.0%
	26–30	62	8.6%
	31 and above	72	10.0%
Mobile Phone Use (in hours)	1–2	19	2.6%
	3–4	171	23.7%
	5–6	271	37.5%
	7–8	262	36.2%
Living Situation	On-campus hostel	10	1.4%
	Off-campus with family	586	81.1%
	Off-campus with roommates	52	7.2%
	Alone	75	10.4%

**Table 2:** Summary of Constructs.

Constructs	Mean (M)	SD	Severity Threshold (Cut-Off)	% Above Threshold
Nomophobia (NMP-Q total)	96.4	22.7	Severe > 100	35%
Depression	13.8	8.2	Severe ≥ 21	18%
Anxiety	12.9	7.6	Severe ≥ 15	22%
Stress	15.5	8.9	Severe ≥ 26	14%
Mindfulness (MAAS)	3.58	0.74	—	—

**Table 3:** Convergent Validity and Reliability.

Factors	FL	CA	CR	AVE	Factors	FL	CA	CR	AVE
Anxiety		0.915	0.919	0.662	Information		0.871	0.893	0.719
ANXI1	0.753				INFO1	0.894			
ANXI2	0.831				INFO2	0.870			
ANXI3	0.835				INFO3	0.779			
ANXI4	0.854				INFO4	0.844			
ANXI5	0.834				Mindfulness		0.979	0.980	0.774
ANXI6	0.797				MIND1	0.826			
ANXI7	0.784				MIND10	0.894			
Communication		0.878	0.908	0.612	MIND11	0.846			
COMM1	0.799				MIND12	0.898			
COMM2	0.819				MIND13	0.916			
COMM3	0.702				MIND14	0.878			
COMM4	0.826				MIND15	0.916			
COMM5	0.722				MIND2	0.868			
COMM6	0.817				MIND3	0.874			
Connectedness		0.887	0.949	0.687	MIND4	0.881			
CONN1	0.814				MIND5	0.875			
CONN2	0.849				MIND6	0.873			
CONN3	0.903				MIND7	0.885			
CONN4	0.74				MIND8	0.889			
CONN5	0.831				MIND9	0.87			
Convenience		0.870	0.888	0.656	Stress		0.886	0.898	0.594
CONV1	0.767				STRS1	0.722			
CONV2	0.815				STRS2	0.788			
CONV3	0.87				STRS3	0.819			
CONV4	0.796				STRS4	0.828			
CONV5	0.799				STRS5	0.807			
Depression		0.886	0.889	0.595	STRS6	0.695			
DEPR1	0.74				STRS7	0.723			
DEPR2	0.805								
DEPR3	0.787								
DEPR4	0.78								
DEPR5	0.81								
DEPR6	0.715								
DEPR7	0.76								

Note: FL: Factor loading; CA: Cronbach alpha; CR: Composite reliability; AVE: Average variance extracted.

**Table 4:** HTMT Ratio—Discriminant Validity and Reliability.

	ANXI	COMM	CONN	CONV	DEPR	INFO	MIND	STRS
ANXI								
COMM	0.225							
CONN	0.248	0.678						
CONV	0.313	0.654	0.786					
DEPR	0.786	0.251	0.339	0.330				
INFO	0.301	0.637	0.815	0.820	0.318			
MIND	0.369	0.119	0.149	0.232	0.456	0.191		
STRS	0.880	0.189	0.211	0.313	0.795	0.265	0.407	

Note: ANXI: Anxiety; COMM: Communication; CONN: Connectedness; CONV: Convenience; DEPR: Depression; INFO: Information; MIND: Mindfulness; STRS: Stress.

**Table 5:** Fornell Larcker Criterion—Discriminant Validity and Reliability.

	ANXI	COMM	CONN	CONV	DEPR	INFO	MIND	STRS
ANXI	0.813							
COMM	0.219	0.782						
CONN	0.231	0.592	0.829					
CONV	0.277	0.562	0.676	0.810				
DEPR	0.714	0.228	0.304	0.288	0.772			
INFO	0.271	0.537	0.720	0.761	0.275	0.848		
MIND	−0.359	−0.126	−0.148	−0.222	−0.428	−0.183	0.880	
STRS	0.800	0.173	0.189	0.270	0.704	0.230	−0.391	0.771

Note: ANXI: Anxiety; COMM: Communication; CONN: Connectedness; CONV: Convenience; DEPR: Depression; INFO: Information; MIND: Mindfulness; STRS: Stress.

**Table 6:** Model Fit Indices.

	Saturated Model	Estimated Model
SRMR	0.052	0.052
d_ULS	4.272	4.272
d_G	1.586	1.586
Chi-square	6084.242	6084.242
NFI	0.890	0.890

**Table 7:** Hypothesis Testing.

#	Coefficients	*p* Values	95% CI	Decision
**Direct Impacts**				
Nomophobia → Mindfulness	−0.052	0.168	[−0.12, 0.03]	H1 Rejected
Nomophobia → Depression	0.327	0.000	[0.25, 0.41]	H2a Accepted
Nomophobia → Anxiety	0.294	0.000	[0.20, 0.38]	H2b Accepted
Nomophobia → Stress	0.259	0.000	[0.15, 0.35]	H2c Accepted
Depression → Mindfulness	−0.293	0.000	[−0.37, −0.21]	H3a Accepted
Anxiety → Mindfulness	0.006	0.933	[−0.09, 0.10]	H3b Rejected
Stress → Mindfulness	−0.173	0.011	[−0.30, −0.04]	H3c Accepted
**Indirect Impacts**				
Nomophobia → Depression → Mindfulness	−0.096	0.000	[−0.14, −0.06]	H4a Accepted
Nomophobia → Anxiety → Mindfulness	0.002	0.934	[−0.04, 0.05]	H4b Rejected
Nomophobia → Stress → Mindfulness	−0.045	0.020	[−0.08, −0.01]	H4c Accepted

## Data Availability

The raw data supporting the conclusions of this article will be made available by the authors on request.
